# 2ab assembly: a methodology for automatable, high-throughput assembly of standard biological parts

**DOI:** 10.1186/1754-1611-7-2

**Published:** 2013-01-10

**Authors:** Mariana Leguia, Jennifer AN Brophy, Douglas Densmore, Angel Asante, J Christopher Anderson

**Affiliations:** 1 Department of Bioengineering, University of California, Berkeley, CA, 94720, USA; 2 QB3: California Institute for Quantitative Biological Research, University of California, Berkeley, CA, 94720, USA; 3 Physical Biosciences Division, Lawrence Berkeley National Laboratory, Berkeley, CA, 94720, USA; 4 Current address: Department of Electrical and Computer Engineering, Boston University, Boston, MA, 02215, USA

**Keywords:** 2ab reaction, Automated assembly, DNA fabrication, Synthetic biology

## Abstract

There is growing demand for robust DNA assembly strategies to quickly and accurately fabricate genetic circuits for synthetic biology. One application of this technology is reconstitution of multi-gene assemblies. Here, we integrate a new software tool chain with 2ab assembly and show that it is robust enough to generate 528 distinct composite parts with an error-free success rate of 96%. Finally, we discuss our findings in the context of its implications for biosafety and biosecurity.

## Background

Synthetic biology uses a ground-up approach to genetic engineering with a vision to solve the technical and conceptual bottlenecks associated with the field. Standard biological parts have emerged as a mechanism through which researchers can emphasize the engineering component of genetic engineering. To viably implement such a vision, however, a new experimental approach that can systematically identify and optimize biological function is needed. Currently there are no established methodologies to encapsulate biological part function such that meaningful, quantitative predictions about genetic composition can be made a priori. Although in practice post-hoc analyses of projects provide insights into the individual properties of parts, most engineering strategies require either an ad-hoc search of intuitively-chosen constructs or library-based approaches. In the hands of experienced researchers these methods can be quite effective for systems containing up to 6 genes [1]. Nevertheless, beyond this scale these methods appear to be insufficient. We have implemented an alternate approach to experimental design that uncovers optimal solutions to design problems by linking automated DNA fabrication to effective construct screening. In the first step, we use cost effective, fast and scalable DNA fabrication methods to generate large sets of explicit DNA constructs automatically. In the second step, we functionally screen those DNAs via readily available assays that are also automatable, cost effective, fast and scalable. Additionally, we integrate these activities under a software tool chain specifically developed for these types of applications. By linking these functions together into a single process we not only uncover optimal solutions to a design problem, but also encapsulate important data about the parts from which they were composed. In turn, this leads to greater insight and faster optimization.

To illustrate how this alternate approach can be implemented in a synthetic biology lab we have focused on the production of multi-subunit protein complexes. The ability to produce functional protein from recombinant sources is essential in synthetic biology and for the scientific community at large. Nevertheless, producing these proteins quickly and successfully, particularly in the case of multi-subunit complexes, can be challenging. Given an overall lack of tools available to quickly and effectively identify appropriate conditions a priori, production efforts are often inefficient, resulting in poor or failed attempts. In these cases, original constructs need to be modified, which is often costly, labor-intensive and time-consuming, particularly if multiple rounds of modification are necessary. An attractive alternative to sequential, slow, expensive, multiple rounds of modification is the generation, in parallel, of several permutations of a particular genetic architecture, followed by rapid functional screening of those permutations. Currently however, the assembly of long, error-free DNAs of the type required to build complex, tunable genetic circuits is the primary bottleneck in the field.

In recent years several DNA assembly strategies have been described, including Gibson, SLIC, Golden Gate, and various BioBrick approaches (for reviews see [2-5]). Each method has a range of advantages and disadvantages, and thus, different construction scenarios are best served by different technologies. Some of these methods can be used to assemble DNAs simultaneously. However, in the context of the design paradigm described here, a key parameter to consider is whether a particular strategy can be implemented in an automated platform that is amenable to scaling. Clearly, some chemistries will automate better than others. Thus, indentifying DNA fabrication protocols that are compatible with automation and scaling is an essential step towards easing the construction jam. Furthermore, quick functional screens of assembled permutations, via established assays that can also be automated and scaled, is essential for the isolation and control of most, if not all, of the variables affecting a particular project.

Here, we describe the construction of a set of 528 distinct composite devices that sample a large combinatorial space. Specifically, every device is a bi-cistronic operon constructed out of 8 standard basic parts. Operons encode two separate protein fusions containing an epitope tag either at the N- or C- terminus that is used to establish function. With support from software tools specifically developed for these types of DNA fabrication experiments [6,7], we have generated all constructs automatically, using a methodology called 2ab assembly. 2ab assembly is a single iterative process, based on the BglBricks standard [8], that is amenable to high-throughput automation and scaling [7]. Like other BioBricks™-based schemes, the BglBricks standard is based on unique restriction enzyme sites that flank the part (BglII at the 5^′^ end and BamHI at the 3^′^ end) and generate compatible cohesive ends that can be joined back together. When two parts are joined using simple restriction digestion and ligation reactions, the resulting composite part also contains unique BglII and BamHI sites flanking the 5^′^ and 3^′^ ends, respectively, as well as a small scar sequence encoding Gly-Ser separating input parts. As we show here however, 2ab assembly is not solely dependent on the BglBricks standard. In fact, 2ab assembly is flexible enough to accommodate alternate assembly standards as well. Using simple ELISA assays we have probed a variety of parameters that influence protein expression in functional form. Further, we surveyed and identified optimal architectures that result in the expression of functional protein complexes. We show that 2ab assembly is robust enough to generate hundreds of large error-free DNAs of the type required in a single experiment within the paradigm of synthetic biology with a 96% success rate.

## Results

### Selection and design of an appropriate DNA assembly set: bi-cistronic operons of Mediator complex for protein production

To test this new experimental approach we set out to generate a large set of DNA constructs that would allow us to measure the robustness of our assembly methodology. Further, we wanted to make use of assembled DNAs to address a challenging biological problem of significant current relevance. The expression and purification of multi-subunit protein complexes in soluble form is one such challenge, particularly because there are no tools that can be used at the outset of an experiment to select strategies that will yield successful results. Protein production is influenced by multiple variables, including expression host, construct composition and architecture, timing, rate, and temperature of expression, purification method, and so on. In the case of multi-subunit complexes, potential challenges can be exacerbated because individual subunits may require co-expression and/or independent regulation of expression. Thus, it may be necessary to consider separate control of additional variables, such as expression timing, rate and stoichiometry, which can further complicate a project.

As proof-of-concept for our 2ab assembly methodology we put together a set of 528 unique plasmids encoding permutations of bi-cistronic operons that could be used to express, identify and purify distinct epitope-tagged proteins known to form a stable complex in *E. coli*. The proteins selected were Med7 and Med21, two subunits of the *S. cerevisiae* Mediator complex. Mediator is a large protein machine (more than 1 MDa in size, containing 20+ independent subunits) that functions as a key regulator of eukaryotic transcription [9,10]. Despite its importance, however, the size and complexity of Mediator pose specific research challenges. Thus, tools that can help address such difficulties are useful to begin to address existing obstacles. Med7 and Med21 were specifically selected as a suitable pair for this assembly because they have been previously purified in *E. coli* and because *E. coli*-expressed Med7 and Med21 can form stable hetero-dimers [11]. Thus, such an assembly set would allow us to test the functionality of our constructs post-assembly in a variety of ways: for example, by measuring relative protein expression profiles of individual subunits; or by measuring the degree of protein-protein interaction between separate subunits.

**Figure 1:**
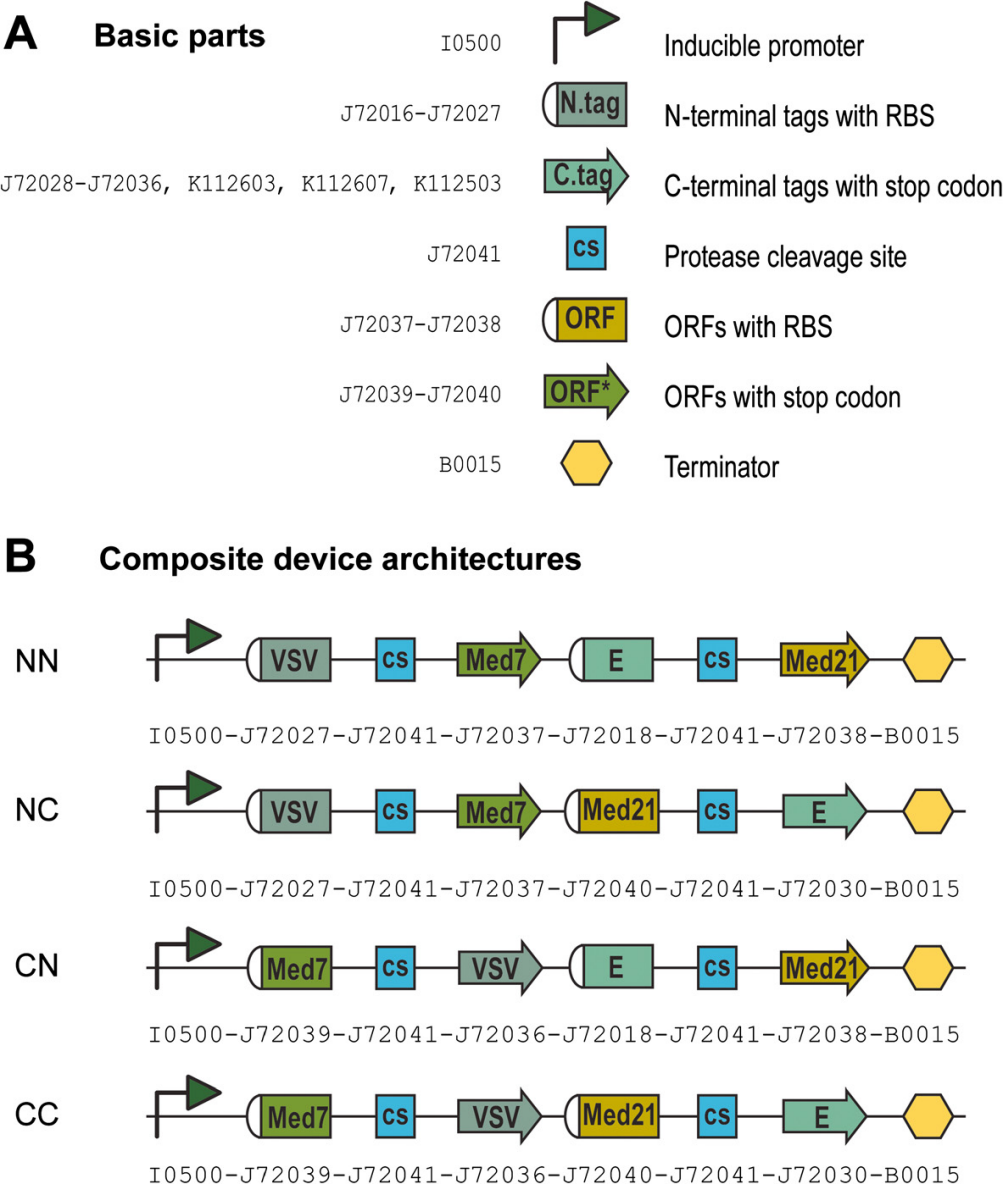
**Assembly of bi-cistronic operons. **(**A**) Basic parts used in the assembly. ORFs and tags were synthesized as either N- or C-terminal sets. N-terminal sets contain RBSs as a component of the part, while C-terminal sets contain stop codons at the end of the part. (**B**) Sample combinatorial architectures for a set of constructs containing two ORFs (Med7 in the first position and Med21 in the second position) tagged at the N- or C-terminus with different tags (VSV-tag for Med7 and E-tag for Med21). Every operon is composed of 8 basic parts. For this arrangement, 4 different construct architectures are possible: in the NN configuration both ORFs are tagged at the N-terminus; in the NC configuration the first ORFs is tagged at the N-terminus and the second ORF is tagged at the C-terminus; in the CN configuration the first ORFs is tagged at the C-terminus and the second ORF is tagged at the N-terminus; in the CC configuration both ORFs are tagged at the C-terminus.

We tagged each Mediator subunit with a small epitope that would enable a variety of downstream assays, including but not limited to, detection of individual subunit expression levels and protein-protein interactions. We used 12 different tags (6X-His, Avi, E, FLAG, HA, HSV, Myc, S, Strep, T7, V5 and VSV) (full details provided in Additional file 1) for which there are commercially-available antibodies (listed in Additional file 2). We varied the location of the tag relative to the protein ORF (either at the N- or the C-terminus) and we sampled all possible architectures: both subunits tagged at the N-terminus [NN] or C-terminus [CC]; first subunit tagged at the N-terminus and second subunit tagged at the C-terminus [NC]; and vice-versa [CN] (Figure 1). In each case, tags were separated from their corresponding ORFs by a TEV protease cleavage in order to facilitate tag removal if desired. Expression of every operon was placed under the control of an arabinose-inducible Pbad promoter and terminator. All bi-cistronic operons were composed of 8 basic parts each: a promoter to drive expression, followed by the first ORF (consisting of 3 basic parts: Med7 and one epitope tag, separated by a TEV cleavage site), followed by the second ORF (also consisting of 3 basic parts: Med21 and one epitope tag, separated by a TEV site), followed by a terminator. The Eugene programming language, which was specifically created for the specification of synthetic biological designs from a collection of individual standard parts [6], was used to generate our assembly set given a defined set of composition constraints. These constraints are declarative rules that either prevent or ensure the appearance of specific tag combinations in a construct. The general bi-cistronic operon architecture can then be permuted while adhering to these constraints. Given 12 tags and 4 possible tagging architectures (NN, NC, CN and CC), the number of bi-cistronic operons needed to sample the entire combinatorial space was 528.

All 528 unique bi-cistronic operons were assembled from a total of 31 basic parts (Additional file 1). Basic parts encoding tags and ORFs were gene synthesized in two versions in order to simplify construction: either with RBSs and start codons at the beginning, or with stop codons at the end. Tags in the first group, referred to as N-terminal tags, were designed to sit at the N-terminus of Mediator subunits. They included an RBS (determined using the RBS calculator algorithm [12] set at a target translation rate of 10,000), an ATG start codon for initiation of translation, and lacked an in-frame stop codon at the end. Tags in second group, referred to as C-terminal tags, were designed to sit at the C-terminus of Mediator subunits. They did not include an RBS or a start codon, but they did contain an in-frame stop codon at the end. Mediator subunits were similarly synthesized. N-terminal ORFs accepted N-terminal tags and did not include an RBS, but did contain an in-frame stop codon. C-terminal ORFs accepted C-terminal tags and included an RBS (determined as above), but lacked a stop codon at the end. Both Med7 and Med21 basic parts were made as truncations that express well in *E. coli* and retain their ability to interact with each other [11]. Med7 was truncated N- and C-terminally and included amino acids 102–205, while Med21 was truncated C-terminally only and included amino acids 1–132. The protease cleavage site part was gene synthesized without start or stop codons because it would separate tags from their corresponding Mediator subunits.

Assembly of all target plasmids was completed in 3 stages of 2ab assembly, according to an assembly tree generated by the AssemblyManager software tool, using a robotics platform containing a Biomek 3000 liquid handling robot [7,13]. AssemblyManager examined the set of devices to be constructed as a group and created an optimal assembly tree. Optimality was defined by a minimal depth tree, where the stages indicated sets of parallel 2ab assembly reactions. The number of steps (a single 2ab assembly reaction) in the tree was also minimized. Steps were further reduced by sharing intermediate reactions. The tree was then post-processed to assign antibiotic resistance markers to input nodes. In the event of a conflicting antibiotic assignment, the tree was modified to accommodate the correct resistance at the expense of the overall assembly cost. A set of heuristics were used to accomplish this process [13]. Specifically for this assembly, we used 31 basic parts to make 56 composite parts (made of 2 basic parts each) in stage 1. In stage 2, we used the parts made in stage 1 to assemble 48 composite parts (made of 4 basic parts each). In the final stage, we used the parts made in stage 2 to assemble 528 composite parts (made of 8 basic parts each). The target set of 528 plasmids contained a total of 3696 junctions between parts. However, most of these junctions were not unique, and therefore in practice, only 632 total junctions were needed to complete the set.

### Definition of 2ab assembly

2ab assembly is a DNA fabrication methodology that uses 2ab reactions to build progressively more complex composite parts starting from basic standard biological parts. 2ab reactions proceed in iterative cycles of restriction digestion, ligation, transformation and selection of desired products. The reaction enables ligation by double antibiotic selection of 5^′^ and 3^′^ parts, designated as “lefty” and “righty” respectively, located on two different assembly vectors.

The core of 2ab assembly is a set of highly engineered plasmids containing two different antibiotic resistance genes (from a total of three: A = ampicillin, C = chloramphenicol, K = kanamycin) separated by restriction site. Given these three antibiotics, six different combinations of assembly vectors are possible: AC, CK, KA, AK, KC and CA. For a given vector pair to work properly during 2ab assembly, the choice of vector pair is pre-determined such that, once two plasmids recombine to form new architectures, desired child products can be selected away from undesired products, as well as from parents, using differential antibiotic selection.

**Figure 2:**
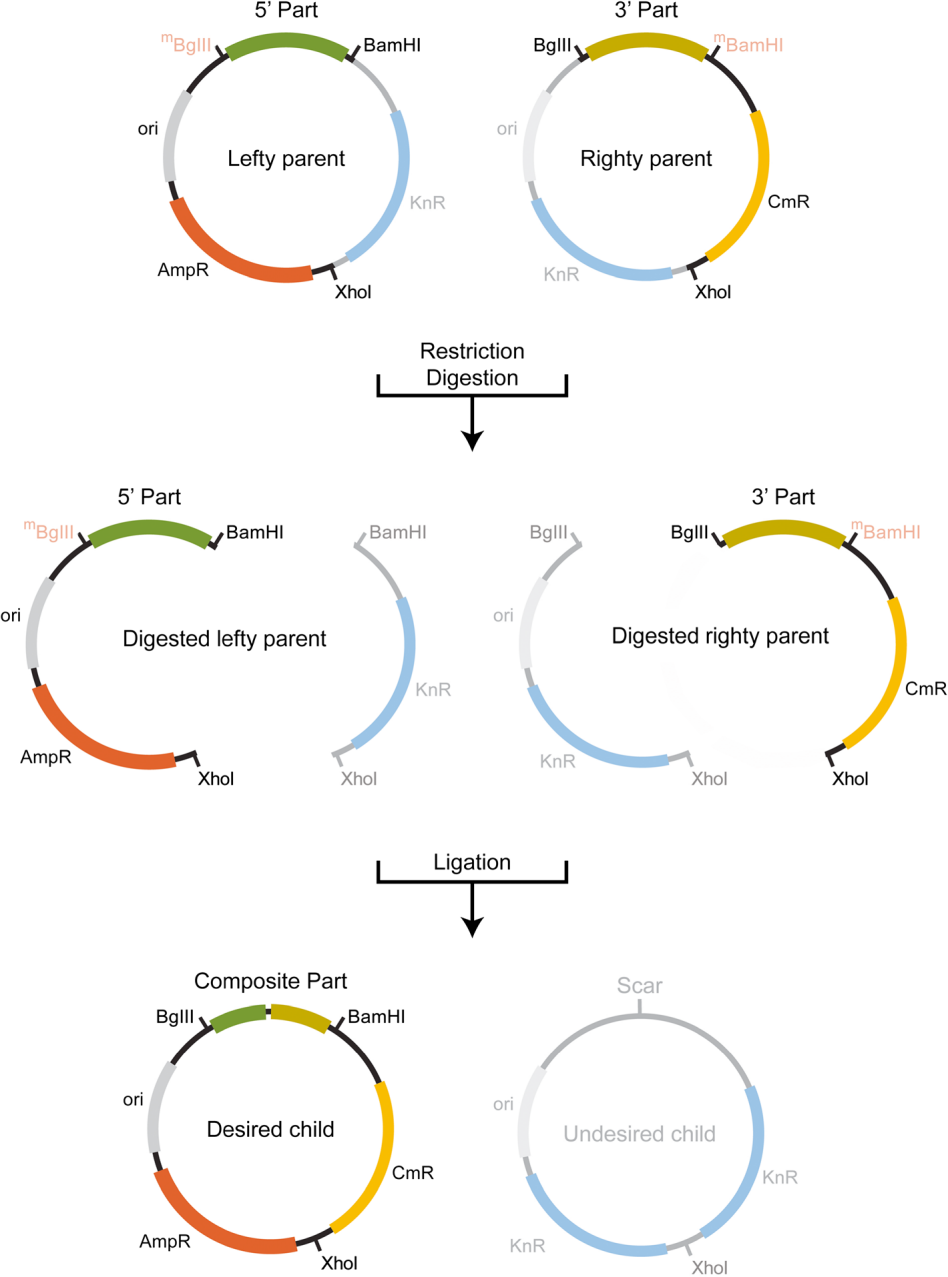
**The 2ab reaction. **These are carried out *in-vitro* and used to make junctions between parts located on different assembly vectors. The choice of vector pair for each junction is determined by an assembly tree generated by AssemblyManager. AssemblyManager takes into account antibiotic resistance markers on both lefty and righty input plasmids and generates a binary tree containing legal lefty and righty pairs. In practice, lefty and righty elements are made by harvesting plasmids from *E. coli* strains that specifically methylate either BglII (part J72015) or BamHI (part J72015) restriction sites (methylated sites are shown in red and grayed over to indicate protection from digestion) [7,8]. Isolated plasmids are then combined in one pot and digested with an enzyme cocktail containing BglII, BamHI and XhoI. Given the methylation state of each plasmid, lefties are cut with BamHI and XhoI, while righties are cut with BglII and XhoI. Following digestion, a ligation reaction recombines all fragments to generate a new composite part with a distinct permutation of antibiotic markers. The desired child product can then be selected away from undesired products, including parent plasmids, by growing it in the appropriate combination of antibiotics. Each new composite part can now be used iteratively in additional 2ab reactions to create progressively more complex parts.

Every assembly reaction is subject to the following three constraints: First, the antibiotic resistance markers on every assembly vector must be distinct from one another (vectors with two AA, CC, or KK genes are not permitted). Second, the resistance marker on the right position of the assembly parent donating the “lefty” part must be the same as the resistance marker on left position of the assembly parent donating the “righty” part. As illustrated in Figure 2, an AK-KC vector pair can be used to assemble parts 1 and 2 together, in that order, provided that part 1, the “lefty” or 5^′^ part, is on AK, while part 2, the “righty” or 3^′^ part, is on KC. Similarly, a CK-KA pair can be used to assemble parts 1 and 2 together, in that order, provided that part 1 is on CK and part 2 is on KA, and so on. The final constraint is that the antibiotic combination associated with the correctly assembled child must be distinguishable from the antibiotic combination associated with the child byproduct, as well as both of the parents. As illustrated in Figure 2, the AK-KC vector pair produces AC and KK combinations when recombined. Both of these are distinguishable from each other and from the parents. Although in theory 2ab assembly can be carried out using a variety of assembly standards, we have developed this methodology for use with the previously described BglBricks standard [8]. Thus, most of the parts in assembly pairs described here are BglBricks parts flanked by BglII and BamHI restriction sites on their 5^′^ and 3^′^ ends, respectively. Further, the two antibiotic markers in every assembly vector are separated by an XhoI site. Modifications to this set-up, where alternate assembly standards like the BBF RFC10 have been used to compare the robustness of BglBricks-based 2ab assembly, are specifically noted throughout.

Prior to the first round of assembly, “lefty” and “righty” parts are defined by specific methylation. For this, lefty and righty plasmids are transformed and harvested from strains of *E. coli* that specifically methylate BglII (part J72015) and BamHI (part J72015) restriction sites, respectively [7,8]. The methylation state of each plasmid, which is different in “lefty” and “right” parts, dictates differential patterns of restriction digestion. In “lefties,” BglII restriction sites are methylated, and thus protected from digestion, while in “righties,” BamHI restriction sites are methylated and protected from digestion.

In practice, 2ab reactions are carried out *in-vitro* in one pot. Following isolation from their corresponding methylation strains, “lefties” and “righties” are combined together and digested with an enzyme cocktail containing BglII, BamHI and XhoI. Given the methylation state of each, “lefty” is cut with BamHI and XhoI, while “righty” is cut with BglII and XhoI. This results in the generation of two linear fragments per plasmid. As illustrated in Figure 2, AK is digested such that the A resistance marker is now on the same fragment of DNA as the “lefty” or 5^′^ part, while the K resistance marker is on its own. Similarly, KC is digested such that the C resistance marker is now on the same fragment of DNA as the “righty” or 3^′^ part, while the K resistance marker is on its own. Following digestion, a ligation reaction recombines all the fragments to generate four distinct possible plasmid architectures. Two are the same as the parent plasmids, while the other two are recombined child products.

Child plasmids are generated when BamHI overhangs of the “lefty” vector and BglII overhangs of the “righty” vector are ligated together on one end, and the two XhoI overhangs are ligated together on the other end. The desired child product contains a new composite part and a new distinct permutation of antibiotic markers (AC in this example), while the child byproduct contains no part and two copies of the remaining antibiotic marker (KK in this example). Given that each possible plasmid architecture contains a different antibiotic combination, desired child products can be selected away from undesired products, as well as from parents, simply by growing them in the appropriate antibiotic combination. Each new composite part can now be used iteratively in additional 2ab reactions to create progressively more complex parts because all the elements necessary for 2ab assembly have been maintained: the new part is still flanked by BglII and BamHI sites on its 5^′^ and 3^′^ ends, respectively; and two antibiotic resistance genes are still present and separated by an XhoI site. In order for the next cycle of 2ab assembly to begin, new composite parts need to be specified as lefties or righties (and methylated accordingly), and compatible assembly vector partners selected. In practice, these choices have been determined by the assembly tree generated using AssemblyManager at the outset of the assembly.

### Screening of 2ab assembly

To measure the robustness of 2ab assembly, every junction was assembled twice, in separate replicate experiments. For every junction made, at least three independent colonies containing putative composite parts were screened. All colonies were subjected to several screens: the first was growth phenotyping under various antibiotic combinations; the second was restriction mapping or colony PCR amplification; and the last was sequencing. These tests were performed in combination in order to ensure proper part assembly at every stage.

In the first screen, single colonies were streaked onto both double (AC, CK or KA, depending on the antibiotic combination marking the new 2ab junction generated) and triple (ACK) antibiotic combinations. Transformants containing correctly assembled parts were able to grow on the appropriate double antibiotic combination (indicating they contained growth markers associated with correctly assembled parts), but were not able to grow on the triple antibiotic combination (indicating possible co-transformation of one of the parent plasmids and/or inappropriate assembly). As shown in Table 1, the average co-transformation rate was about 5% (111 of 2208), indicating that the vast majority of transformants (2097 of 2208) contained growth markers associated with properly assembled parts. Co-transformation rates ranged from 2% for junctions between AK pairs, to 26% for junctions between KC pairs. We routinely observe a wide range of co-transformation rates. The variability is mostly linked to two factors: first, the identity of the antibiotic pair (CK and KC pairs show higher rates of co-transformation than AC, CA, AK and KA pairs when equivalent amounts of plasmid DNA are used); and second, the concentration of plasmid DNA used for transformation (higher amounts of plasmid DNA give rise to higher rates of co-transformation).

**Table 1:** Summary of BglBricks-based 2ab assembly results

**Assembly stage**	**1**			**2**		**3**	**Totals**
junction type	CA	AK	KC	CK	KA	CA	
junctions made	28	14	14	24	24	528	632
colonies screened by growth phenotype	84	42	42	156	156	1728	2208
co-transformants	3	1	11	12	7	77	111
% co-transformation	3.57	2.38	26.19	7.69	4.49	4.46	5.03
colonies screened by mapping or PCR	28	14	14	48	48	622	774
colonies w/ correct map or PCR phenotype	26	13	13	47	48	535	682
colonies w/ questionable map or PCR phenotype	2	1	1	0	0	75	79
colonies w/ incorrect map or PCR phenotype	0	0	0	1	0	12	13
colonies sequenced	9	1	14	9	8	73	114
colonies w/ correct map or PCR sequenced	7	1	13	9	8	65	103
correct sequencing result	7	1	13	9	8	65	103
colonies w/ questionable map or PCR sequenced	2	1	1	n/a	n/a	4	8
correct sequencing result	0	1	0	n/a	n/a	3	4
colonies w/ incorrect map or PCR sequenced	n/a	n/a	n/a	0	n/a	4	4
correct sequencing result	n/a	n/a	n/a	n/a	n/a	0	0
% correct assembly	92.86	100	92.86	97.92	100	95.06	96.45*

*Average of % assembly rates for every junction at various assembly stages.

Transformants showing the correct growth phenotype were further screened either by restriction mapping with BglII, BamHI and XhoI, or by colony PCR amplification. Even though colony PCR amplification is preferable as a screening tool because it does not require extraction of plasmid DNA, which is costly and time-consuming, the sizes of many stage 1 and 2 products could not be clearly resolved from those of their parents with this technique, and thus, were restriction mapped. All products of stages 1 and 2 (104 total) were screened by restriction mapping, while all products of stage 3 (528 total) were screened by colony PCR.

A few clones from every stage of assembly, including those with correct, questionable and incorrect restriction maps or amplicons, were selected for sequencing to verify part identity. As shown in Table 1, about 96% of all colonies screened contained correctly assembly parts. The percentages of properly assembled junctions ranged from 93% for CA and KC pairs, to 100% for AK and KA pairs. The results obtained by sequencing verify that our restriction enzyme mapping and colony PCR screens were stringent enough to assess assembly success accurately. Of the clones showing correct restriction maps or PCR amplicons sequenced (103 total), 100% contained properly assembled parts. Of the clones with questionable maps or PCR amplicons sequenced (8 total), 50% contained properly assembled parts, indicating that we had been conservative in our scoring of proper assembly as assessed by restriction enzyme mapping and colony PCR. Of the clones with incorrect restriction maps or PCR amplicons sequenced (4 total), none contained properly assembled parts.

To further test the robustness of 2ab assembly, we compared the success rate obtained by BglBricks-based 2ab assembly to that obtained using a different assembly standard also in the context of 2ab assembly. For this, we chose the original BBF RFC10 standard described by Knight [14], which uses SpeI and XbaI restriction sites flanking the 5^′^ and 3^′^ ends of parts, respectively. Specifically, we carried out a small assembly using a split version of super folder-GFP [15], expressed under a constitutive promoter, that results in visibly green colonies in *E. coli* when properly assembled. The lefty input part consisted of the first 173 amino acids of sf-GFP driven by the tet promoter, while the righty input part consisted of the last 64 amino acids of sf-GFP. Input parts were subcloned into all six 2ab assembly vectors to test assembly of all junctions. Following assembly, lefty and righty input parts are separated by a small scar sequence (ACTAGA) encoding Thr-Arg, which does not interfere with the fluorophore. As shown in Table 2, the average co-transformation rate was about 14% (174 of 1199), indicating that the vast majority of transformants (1025 of 1199) contained growth markers associated with properly assembled parts. Co-transformation rates ranged from 12% for junctions between KA pairs, to 19% for junctions between CK pairs. Proper assembly was further assessed by counting green colonies. About 92% of colonies screened (3221 of 3486) were positive for GFP expression. The percentages of properly assembled junctions ranged from 91% for CK pairs, to 95% for AC pairs. To ensure that we were not under-reporting the number of correct assemblies, we sequenced the assembly products extracted from 26 white colonies. Of these, none contained properly assembled parts, indicating that our screens were robust enough to assess assembly success accurately.

**Table 2:** Summary of BBF RFC10-based 2ab assembly results

**Assembly stage**	**1**						**Totals**
junction type	CA	AK	KC	CK	KA	AC	
total junctions made	1	1	1	1	1	1	6
colonies screened by growth phenotype	200	198	201	200	199	201	1199
co-transformants	29	26	27	37	24	31	174
% co-transformation	14.50	13.13	13.43	18.5	12.06	15.42	14.51
total colonies counted	580	1011	274	366	688	567	3486
white colonies	45	83	24	34	48	31	265
green colonies	535	928	250	332	640	536	3221
total colonies sequenced	5	6	5	2	7	1	26
white colonies sequenced	5	6	5	2	7	1	26
correct sequencing result	0	0	0	0	0	0	0
green colonies sequenced	0	0	0	0	0	0	0
correct sequencing result	n/a	n/a	n/a	n/a	n/a	n/a	0
% correct assembly	92.24	91.79	91.24	90.71	93.02	94.53	92.40

### Characterization of errors

In the process of assessing the robustness of our 2ab assembly methodology we identified three main sources of error. The first was co-transformation of plasmids, which has been briefly mentioned above. Co-transformation results in colonies that are able to grow in 2ab combinations marking the newly assembled junction, but that may or may not contain properly assembled parts. Instead, these colonies contain antibiotic resistance markers associated with correctly assembled junctions in one or more plasmids that may or may not include the assembled plasmid of interest. Although there was a slight qualitative correlation between co-transformation and certain antibiotic combinations (CK and KC pairs showed higher rates of co-transformation than AC, CA, AK and KA pairs when equivalent amounts of plasmid DNA are used) there was no correlation to part size or part source. Instead, co-transformation was directly linked to the concentration of plasmid DNA used in transformation reactions. Higher amounts of plasmid DNA give rise to higher rates of co-transformation, thus, a simple way to reduce them is to dilute the amount of DNA used for transformation. Although co-transformation rates were not high enough to present a problem in our assemblies, these colonies can be easily screened and eliminated by simultaneously re-streaking on double and triple antibiotic combinations. Colonies containing properly assembled parts will be able to grow on two antibiotics marking the newly assembled junction, but they will not be able to grow on all three. Any colony that grows on triple antibiotic combinations should be discarded.

A second, less common source of error (12 instances out of 774), results in colonies that exhibit the appropriate biotope, but that contain unrecognizable plasmids. In every one of these cases, the failure could be correlated back to poor quality parent input plasmid DNA and corrected by re-extracting the input plasmid.

The last source of error resulted in the production of junctions lacking whole input parts. In total, we identified 80 instances of BglBricks-based 2ab assembled junctions, and 9 instances of BBF RFC10-based 2ab assembled junctions, containing assembly scars but lacking either the entire lefty or righty input part. In the case of BglBricks-based assembly, empty junctions arise as a result of incomplete methylation of parent plasmid DNA. When input plasmids are incompletely methylated they are subject to part loss during digestion steps with BglII and BamHI, which effectively eliminates the input part from the reaction but maintains the vector backbone available for 2ab assembly. In the case of BBF RFC10-based assembly, empty junctions arise as a result of incomplete heat-inactivation of restriction enzymes following digestion steps, which has the same effect as incomplete methylation of parent input DNA. Given that partial methylation is a limitation of the methylation strains themselves (which have the potential to be significantly improved), rather than of 2ab assembly *per se*, our reported success rate of 96% is a conservative estimate of the actual rate, indicating that our technology is indeed robust enough to construct large error-free DNA sets with a very high success rate.

### Screening the functionality of assembled bi-cistronic operons

Addressing the bottleneck around DNA fabrication is only the first step towards developing high-throughput solutions in synthetic biology. Thus, in addition to showing that our DNA assembly methodology is sufficiently robust to build large error-free DNAs with a ~96% success rate, we were interested in testing the functionality of assembled constructs. To do this in a cellular context, we induced expression and used indirect ELISA assays to quantify steady-state relative protein expression levels from every epitope-tagged Mediator subunit in all 528 bi-cistronic operons (entire data set is provided in Additional files 3 and 4). For a select subset of operons, we also used sandwich ELISA assays to probe protein-protein interactions and verify functionality of individually expressed proteins (data not shown). For the purposes of this assembly however, it was much more important to have a complete data set that would allow us to comparatively asses operon functionality (quantified by measuring relative protein expression levels via indirect ELISAs), than to asses individual subunit functionality (quantified by measuring protein-protein interactions via sandwich ELISAs). Given these priorities, and given the large number of operons in the assembly set (528 plasmids, assayed for two tags each, in triplicate), we used indirect ELISAs, which are simpler and faster to carry out, to generate a complete data set that allowed us to make side-by-side comparisons and predictions.

**Figure 3:**
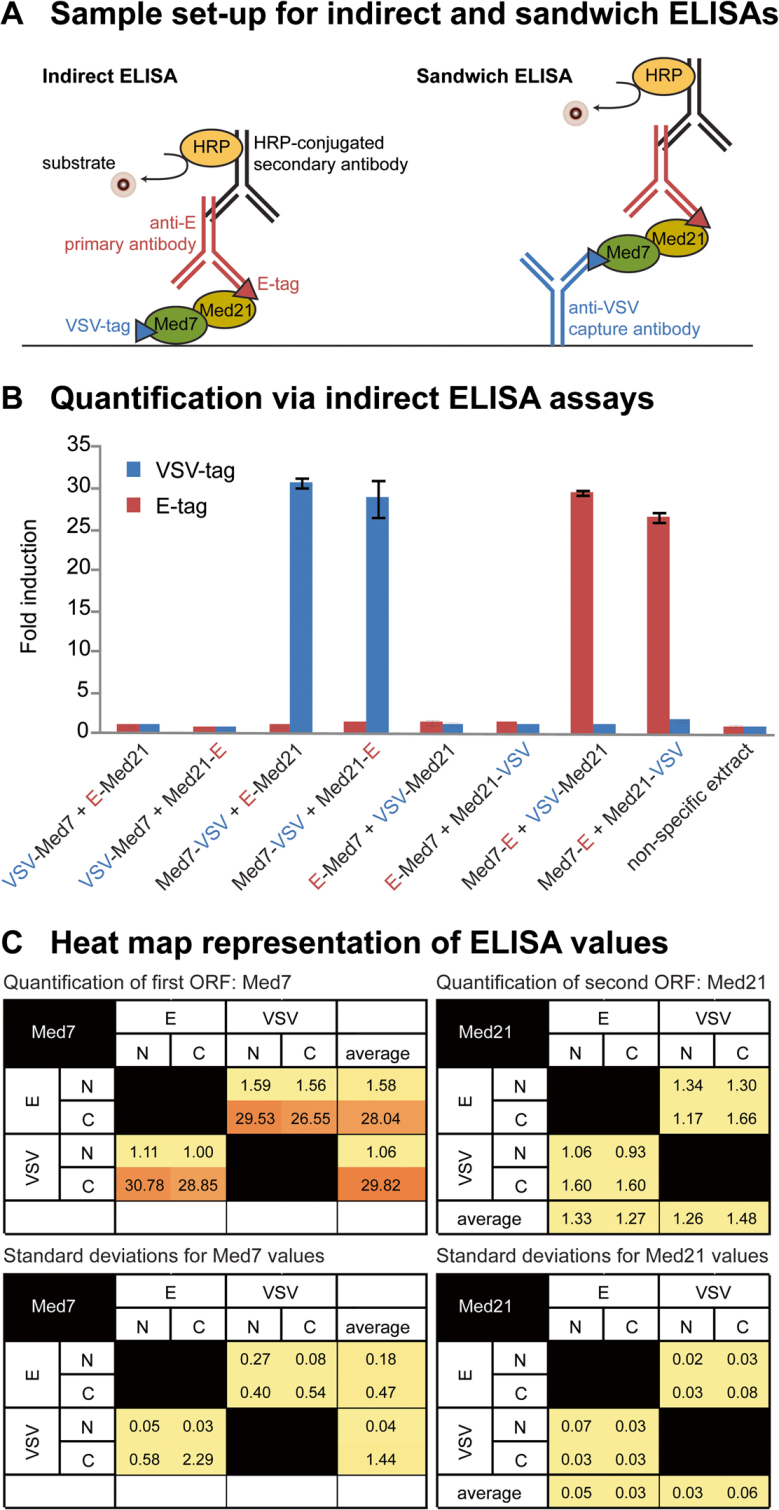
**Quantification of protein expression from differentially tagged ORFs. **(**A**) Sample set-up for indirect and sandwich ELISA assays used to quantify protein expression of individual subunits and protein-protein interactions, respectively. (**B**) Indirect ELISA data for a representative set of 8 constructs sharing VSV and E tags. For any two tags, 8 different architectures are possible. In the first four constructs VSV-Tag (in blue) is fused to the first ORF (Med7) and E-Tag (in red) to the second ORF (Med21). In the next four constructs, which are “reciprocal” architectures, VSV-Tag is fused to the second ORF (Med21) and E-Tag to the first (Med7). Constructs with tags in common are assayed in tandem, replicate experiments. Reported values are normalized for protein concentration and background. Control experiments include antibody drop-outs (not shown) and protein extracts lacking epitope tags. (**C**) ELISA values plotted into “heat maps” to better visualize patterns of expression. The top two maps show averaged values from replicate experiments, while the bottom two maps show the corresponding standard deviations. Maps on the left show Med7 expression patterns from the first ORF position (on the “y” axis) and should be read from left to right. Maps on the right show Med21expression patterns from the second ORF position (on the “x” axis) and should be read from top to bottom. Red and yellow colors indicate high and low relative expression levels, respectively.

To assay the operons, they were first sorted by tag and grouped together such that those with tags in common were assayed in tandem in replicate experiments. Figure 3 shows indirect ELISA data for a representative set of constructs sharing VSV and E tags. Given that each operon contains two different tags, and given 4 possible tagging architectures (NN, NC, CN and CC), every pair of tags appears in 8 different constructs. In the first four constructs VSV-Tag is fused to the first ORF (Med7) and E-Tag to the second ORF (Med21). In the last four constructs, which are “reciprocal” architectures, VSV-Tag is fused to the second ORF (Med21) and E-Tag to the first (Med7). The results show that varying the location of the tag from the N- to the C-terminus of an ORF can have a dramatic effect on protein expression levels. In this set, Med7 expressed well when tagged at the C-terminus, but significantly less so when tagged at the N-terminus. In turn, Med 21 expressed at lower levels than Med7, either when tagged at the N- or C-terminus.

High-throughput approaches such as these enable rapid processing of large numbers of samples in parallel, which in turn allows the simultaneous visualization of multiple sets of data such that patterns can become readily apparent. From these patterns, many of the ways in which multiple variables influence experimental outcomes begin to emerge. To test the hypothesis that varying the location of the tags would have a more significant effect on protein expression levels than varying the identity of the tag itself, we plotted protein concentration values obtained via ELISAs into “heat maps” that included all possible tags and tagging combinations (Additional file 3). In the context of a heat map, the pattern of expression for Med7 becomes particularly obvious. Regardless of which tag was used, Med7 expression levels were significantly higher when the protein was tagged C-terminally. In some instances, Med7 expression levels in excess of 55 fold over background were observed. An exception to this pattern was FLAG, which resulted in expression levels close to background in all architectures tested. The pattern of expression for Med21 was a bit less obvious than that for Med 7 because overall Med21 expressed at lower levels. In cases where patterns were discernible, as in the case of tag combinations between Avi, HSV and T7, for example, it was apparent that Med21 expression tends to be higher when the protein is tagged C-terminally.

**Figure 4:**
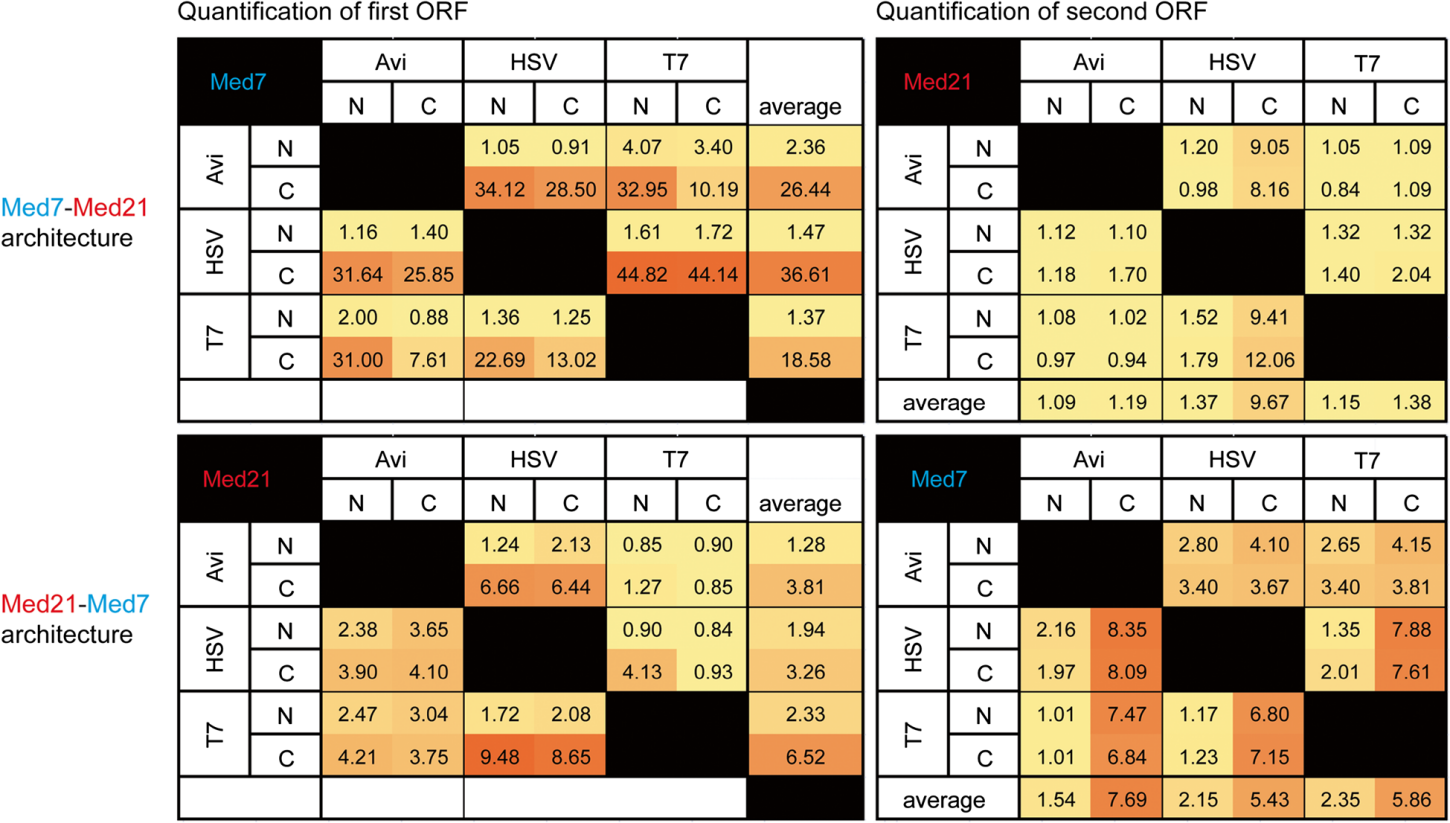
**Heat maps illustrating the effect of switching the location of specific ORFs relative to the driving promoter. **Maps include all possible tagging combinations for 24 bi-cistronic operons sharing tags Avi, HSV and T7. The top two maps illustrate patterns of expression for a set of constructs where Med7 and Med 21 are located in the first and second ORF positions, respectively. The bottom two maps illustrates patterns of expression for the same set of epitope tags only that Med7 and Med 21 are now located in the second and first ORF positions, respectively. Maps on the left quantify expression levels for the first ORF (in the “y” axis) and should be read from left to right, whereas maps on the right quantify expression levels from the second ORF (in the “x” axis) and should be read from top to bottom. As before, red and yellow colors indicate high and low relative expression levels, respectively.

Given the observed patterns we hypothesized that the location of an ORF with respect to the driving promoter would also influence expression, and specifically, that expression from ORFs located proximally to the promoter would be higher than from ORFs located distally. To test this we assembled a small sub-set of 24 bi-cistronic operons where we switched the location of the two ORFs, placing Med21 in the first position, and Med7 in the second. We selected three tags (Avi, HSV and T7) that had shown discernible patterns in the first assembly and tested them as before. Figure 4 shows that, while the tag location patterns remained the same (both ORFs expressed better when tagged C- rather N-terminally), switching the location of the ORFs equalized protein expression significantly. In the first assembly, where Med 7 was in the first ORF position and Med21 in the second ORF position, the fold difference between the two highest expressing architectures in the sub-set (Med7 at 44.82 and Med21 at 12.06) was ~33 fold. In the second assembly, where Med 21 was in the first position and Med7 the second, the fold difference between the two highest expressing architectures in the sub-set (Med21 at 9.48 and Med7 at 8.35) decreased to ~1fold. Control of this variable alone can have a significant impact on experimental outcomes, which is particularly noteworthy given that producing protein in stoichiometric equivalences that enable formation of functional soluble complexes is often more important than producing large quantities of non-functional single subunits. Taken together, our results confirm that recombinant protein expression is influenced by multiple distinct variables that correlate with specific sequences of DNA at specific locations in a plasmid. By enabling the rapid and effective construction of plasmid variants, 2ab assembly enables access and further control over various experimental conditions which, in turn, facilitate tuning of gene expression circuits used in synthetic biology.

## Discussion

We share the vision of synthetic biology as a field dedicated to solving the technical and conceptual bottlenecks of genetic engineering. Although we currently face several challenges, it is clear that many of these obstacles can be attributed either to an insufficient survey of the design space or to a lack of knowledge about the molecular and cellular functions of added components. Both of these problems can be addressed by robust, cost-effective, rapid, and outsourced DNA fabrication platforms that lower barriers to fabrication and streamline assembly. Basic standard biological parts, such as BioBricks™, as well as standard assembly strategies to join them together head-to-tail, have emerged as a way to deal with the potential cloning problems associated with the uniqueness of each DNA sequence. The rationale behind these approaches is that by standardizing parts and part junctions to conform to a particular set of rules, considerations of design and function can be separated away from those pertaining to assembly. Furthermore, because the products generated via these methods are themselves standardized parts, a single, iterative, standard assembly reaction can be used to concatenate parts together to form progressively more complex genetic devices. Thus, the problem of composition can be pursued without consideration for how the DNAs are assembled together. Our results indicate that it is possible to robustly and effectively automate the fabrication of hundreds of genes using BioBricks™-based approaches. Several alternate chemistries that can also be used as standard fabrication methods, including SOEing, SLIC, CPEC, Golden Gate Shuffling, and the isothermal method reported by Gibson [16-21], to name a few, have been recently developed. We anticipate that many of these could be similarly automated, and thus, that specific types of experiments will be better served by different assembly methodologies.

Although we currently lack specific benchmarks to compare our results to those obtained using the alternate chemistries mentioned above, a success rate above 96% for BioBricks™-based approaches is very encouraging, particularly since this is a conservative estimate of efficiency that could be improved upon through minor modifications of the existing set-up. The majority of failures observed were related to colonies containing unrecognizable plasmids, which were always traced back to poor quality input mini-prep DNA. Thus, these errors could likely be mitigated using DNA extraction protocols that perform more consistently. A second source of failure was traced back to incomplete methylation of input plasmid DNA, which gave rise to junctions lacking either lefty or righty parts. These errors can be improved upon with better methylation strains. Several of these strains are currently under development in the lab. In a few instances we also observed that particular combinations of antibiotics performed better than others, which could likely be resolved using DIAL strains or through modifications to regulatory elements, including promoter and/or RBS mutations.

Automated assembly enables cumulative information gain in every design cycle by lowering existing barriers to construction and screening. Large numbers of data points reveal patterns that reflect specific underlying properties of the elements contained in a system. Patterns can reflect several distinct variables. In the examples presented here, two of the variables observed to have a significant effect on relative protein expression levels were the location of an epitope tag within an ORF, and the location of that ORF relative to its driving promoter (first or second position in a bi-cistronic operon). As demonstrated previously, other variables can also be used to manipulate expression systems, including RBS and/or promoter mutations [22]. Further, these variables can be used to test whether an engineered device is behaving in a manner that is quantitatively consistent with a particular model. Despite the fact that observed patterns can be useful tools for the interpretation of results, particularly because they enable informed decisions about how the system should be manipulated in subsequent cycles, these trends can rarely be reliably mapped onto specific biophysical parameters. Thus, one of the main challenges we still face is how to encapsulate the root of observed patterns. Ideally, combinatorial sets could be used to quantify parameters that reflect inherent properties of a particular part. As shown with the test sets described here, the patterns observed had, at the very least, qualitative utility for deciding whether expression levels of independent ORFs should be increased or decreased, and by how much, in order to obtain stoichiometric equivalences. Although it is tempting to try to directly encapsulate measurements like ELISA or fluorescence reads as relative measures of particular biophysical values, like transcription, translation or folding rates, we want to root our theory of encapsulation in real numbers that can be directly mapped to actual biophysical parameters reflecting molecular function, such as a molecule’s K_m_, K_cat_, folding rate, etc. Ironically perhaps, the most central parameter that we currently manipulate in synthetic biology, expression level, cannot be easily characterized or encapsulated in terms of such parameters. To get there, one would be fully justified to express a sigma70 binding constant for a promoter, for example. Nevertheless, that alone would not encapsulate everything the promoter could do. In practice, transcription rates would depend on many factors simultaneously exerting an effect within the context of the cell, including load and stress, to name just a few. Although reading trends and patterns within a well-defined context is clearly useful, we still have a long way to go before we can encapsulate information that seeks to define narrow parameters, like whether a protein prefers to be tagged at the N- or C-terminus, or whether using a particular tag will work on a particular percentage of proteins or times. Given that undoubtedly these are statements about molecular function that are related to folding kinetics, it will be necessary to develop theory that will eventually enable us to properly measure these types of parameters, such that they can be reported in biophysically-justifiable terms.

Here we have successfully automated fabrication and characterization of a large set of DNAs based on standard biological parts. Although the process was neither easy nor straightforward, it is clear that it was doable. In fact, the theory of how to do this is simple, and implementation difficulties stem from the fact that it is challenging to automate this type of fabrication and characterization in a single, streamlined liquid handling platform, not from any intrinsic complications associated with the building of large sets of DNAs on automated platforms. To do this well in practice, it would need to be implemented in the context of a centralized DNA fabrication facility that has an assembly line of robots dedicated to specific tasks along the assembly process. Given advances in acoustic liquid handling and microfluidics, more streamlined hardware solutions may soon be available. In the mean time, we should state that the most striking observation coming out of these studies was the lack of appropriate software solutions available to integrate automation tools in a traditional academic laboratory. Electronic information held in our lab, like in many labs, is stored within an aggregation of tools that provide reasonable organization for small teams of researchers. These include sequence-management programs like ApE, Google docs for tables of sequences, parts, and samples, and wikis for experimental data. Though tools such as the Registry of Standard Biological Parts and ICE platforms provide excellent solutions for the dissemination of sequence and qualitative usage information, they do not support the full range of data types that must be considered, tracked, and persisted for managing a synthetic biology experiment. For large sets like ours, the data entry process alone becomes unrealistically burdensome when using only these tools, and the lack of cross-tool communication leads to an enormous amount of custom script writing simply to port information from existing tools into the software needed to run the automation hardware. Further, it was clear that user errors, custom notes, and non-standardized formats to freeform documents make conversion into a standardized form difficult and cumbersome. Despite rapid progress in the area of BioCAD tools [6,23-27], basic day-to-day usability and integration into wet-lab workflows remains one of the outstanding challenges to fully realizing the benefits of these tools.

BioCAD-driven wetlab automation has been identified in both academia and industry as the primary driver of new advances in genetic engineering. Considering the biosafety and biosecurity risks associated with DNA fabrication at this level of sophistication, it is important that we begin to draft road maps of the major events in the development of fabrication standards so that proper practices can be anticipated and interventions implemented *a priori*. This is particularly important given the high level of interest that such technologies generate in the DIY community. The question of whether individuals, either acting alone or in small groups, could make use of these advances to set-up clandestine operations is legitimate. In the absence of guarantees the answer seems to be that although not easy at all, it would be possible for teams of 2–3 people to generate a few hundred DNA constructs a month using a similar, minimal automation setup. Clearly, DNA fabrication is just the first hurdle to overcome in a series of required steps before a functional microbial chemical factory is possible. Thus, although initial barriers to entry may not be as high as desirable, increasing the levels of throughput required for success bring about a series of complications that are not easily overcome unless one gets to a much larger operational scale, such as those found in the context of successful and well-capitalized biotech companies. When acting alone, members of the DIY community could potentially work on applications using BioCAD-driven design processes. However, efforts to optimize processes using combinatorial approaches will likely require external support from an angel investor or well-capitalized fund in order to have a meaningful effect. As we demonstrate with our particular data set, full integration of automated tools into a wet-lab environment will require not only hardware, bioware, and software, but also a complete set of tools that are responsive to the practical needs and practices of wet-lab researchers and can manage the information already held in synthetic biology labs. Fortunately, there is great interest within the synthetic biology community to develop community-shared data standards and interoperating procedures [28] that should significantly facilitate progress in moving forward in an ethical and safe manner.

## Conclusions

We have shown the use of an integrated tool chain from abstract design through to fabrication and analysis. The composite devices assembled in our set contained a total of 3696 junctions between parts (out of which 632 are unique) and were assembled in three steps in about 1 month at a cost (without labor) around $4000. From this we have learned that, as it currently stands, the methodology is robust enough to construct hundreds of large error-free DNAs with a 96% success rate. Though there remain many challenges to fully incorporating these automation workflows into the wet-lab work flow, it is clear that this is doable and provides rich information to accelerate the process of engineering specific devices and a critical tool for the development of a robust encapsulation theory.

## Methods

### Materials

Unless otherwise specified, all enzymes were purchased from NEB (Ipswich, MA). Oligos were purchased from IDT (San Diego, CA) and used unpurified. Antibodies were purchased from GenScript (Piscataway, NJ), Bethyl (Montgomery, TX) and Abcam (Cambridge, MA) and used at the dilutions specified below.

### Construction of basic parts

The Registry of Standard Biological Parts (http://partsregistry.org), which is searchable by part number, provides details on the construction of all basic parts used in this study. Additional file 1 lists both part number for the Registry, and ID number for the non-profit plasmid repository Addgene (http://www.addgene.org). Sequences for the oligos needed to gene synthesize basic parts were generated using OligoDesigner (http://andersonlab.qb3.berkeley.edu/andersonSoftware.html). All parts were codon optimized for expression in *E. coli.* Undesirable restriction enzyme sites (BamHI, BglII, EcoRI and XhoI) were removed using GeneDesign (http://baderlab.bme.jhu.edu/gd/). Part identity was confirmed by sequencing, which was outsourced to Quintara (Berkeley, CA). For maintenance, parts were digested with EcoRI and BamHI, and directionally cloned into various 2ab assembly vectors prior to transformation into MC1061 or derivatives [7,8]. All part and vector sequences, as well as physical DNAs and relevant bacterial strains, are available through Addgene.

### 2ab assembly of composite parts

All junctions between parts were generated by 2ab assembly using a minimal hardware platform consisting of a liquid handling robot (Beckman Coulter Biomek 3000 Laboratory Automation Workstation), a plate-spinning bench-top centrifuge (Beckman Coulter Allegra™ 25R) and a 96-well plate thermocycler (MJ Research PTC-200 Peltier Thermal Cycler), with additional support from a software tool called AssemblyManager. Detailed methods for the implementation of 2ab assembly on the Biomek 3000 liquid handling robot have been previously described [7], and sample robot files required to carry out these protocols, as well as the AssemblyManager software tool, are available for download (http://andersonlab.qb3.berkeley.edu/andersonSoftware.html). Briefly, 2ab assembly proceeds in iterative cycles of 3 steps per cycle: in the first step plasmid DNA is digested with restriction enzymes to generate lefty and righty fragments; in the second step DNA fragments are ligated back together to generate new plasmid combinations; and in the last step plasmids are transformation into *E. coli* for selection and screening. For BglBricks-based 2ab assembly, digestion reactions were carried out in one-pot at 37°C for 1 hr, in 96-well PCR plates, in 16 μL volumes containing 10 μL of digestion cocktail (1 μL 10X NEB Buffer 2, 0.5 μL BamHI, 0.5 μL BglII, 0.5 μL XhoI, 7.5 μL water) and 3 μL each lefty and rightly input plasmids. Lefty and righty parts were generated by transforming and harvesting plasmids from BglII- and BamHI-methylating strains (parts J72007 and J72013), respectively [7,8]. These strains are *pir +* MC1061 derivatives that replicate R6K origins found in all assembly vectors. Following digestion, restriction enzymes were heat-killed at 65°C for 20 min. Ligation reactions were carried out at room temperature for 30 min by adding 4 μL of ligation cocktail (0.4 μL NEB Buffer 2, 2 μL 10 mM ATP, 0.5 μL T4 DNA ligase, 1.1 μL water) into each heat-killed 16 μL digestion reaction. Transformation reactions were carried out by adding 30 μL of chemically competent cells, made using either the BglII- or the BamHI-methylating strain, into each 20 μL ligation. Following incubation on ice for 10 min, cells were heat shocked at 42°C for 3 min, and then rescued by adding 100 μL of 2YT and incubating at 37°C for 30 min. Transformations were then plated onto LB agar strips supplemented with appropriate antibiotic combinations (ampicillin at 100 ug/mL, chloramphenicol at 25 ug/mL and kanamycin at 25 ug/mL) using 24-well strip plates (Analytical Sales & Services, cat# 47025). BBF RFC10-based 2ab assembly was carried out similarly to BglBricks-based 2ab assembly, but with minor modifications due to a lack of strains capable of methylating SpeI or XbaI sites. Lefty and righty plasmids were digested separately, at 37°C for 1 hr, in 8 μL volumes containing 5 μL of SpeI and XhoI or XbaI and XhoI digestion cocktail (0.5 μL 10X NEB Buffer 2, 0.25 μL each enzyme, 4 μL water) and 3 μL each lefty or rightly input plasmids. Following digestion, restriction enzymes were heat-killed at 80°C for 20 min. Righty and lefty products were then combined in one pot prior to ligation and transformation, which were carried out exactly as described above.

### Screening of assembled composite parts

Transformants containing putative composite parts were subjected to various screens at every stage of assembly: the first screen was growth under various antibiotic combinations; the second was restriction enzyme mapping or colony PCR amplification; and the last was sequencing. For growth screens, single colonies were spotted onto LB agar plates containing both double (AC, CK or KA) and triple (ACK) antibiotic combinations and grown overnight at 37°C prior to scoring. Transformants with the correct growth phenotype were then screened either by restriction enzyme mapping or by colony PCR. For restriction enzyme mapping, plasmid DNA was extracted using NucleoSpin plasmid columns (Machery-Nagel), either in single or Multi-8 format, according to the manufacturer’s directions, and digested with BglII, BamHI and XhoI digestion cocktail as above. For colony PCR amplification, single colonies were grown overnight at 37°C in 96 well blocks (Analytical Sales & Services, cat# 27P687) containing 1 mL LB liquid medium supplemented with appropriate antibiotics. PCR assays were carried out in 20 μL volumes using 96-well PCR plates. The reaction mixture contained 0.5 units of Taq DNA polymerase, 1.5 mM MgCl, 200 uM dNTPs, 200uM each oligo (ca998: GTATCACGAGGCAGAATTTCAG and G00101: ATTACCGCCTTTGAGTGAGC) and approximately 0.13 μL of saturated bacterial culture, transferred into the 96-well plate containing the reaction mixture using a 96-pin tool (V&P Scientific, cat#246A). Sequencing was outsourced to Quintara (Berkeley, CA).

### Cell lysates

Prior to ELISAs, cell lysates were prepared from induced *E. coli* strain pir-116 containing the composite part of interest. Briefly, frozen glycerol stocks were used to inoculate 1 mL 2YT liquid medium supplemented with antibiotics and grown overnight at 37°C in 96 well blocks. The next morning, saturated cultures were diluted 1:500 into fresh 1 mL aliquots of 2YT liquid medium, supplemented with antibiotics and 0.2% arabinose. These were grown overnight again at 37°C in 96 well blocks. Induced cultures were then pelleted by centrifugation for 5 min at 5400 g and resuspended in 100 μL of Bugbuster HT (Novagen) by vortexing. After a 20–30 min incubation period at room temperature, lysates were cleared by centrifugation for 5 min at 5400 g, and protein concentration determined by BCA assay (Pierce) according to the manufacturer’s directions. Absorbance was measured at 562 nm using a Tecan Safire 2 plate reader. Lysates were diluted 1:10 in TBST (50 mM Tris pH 7.8, 150 mM NaCl, 0.05% Tween-20) prior to use.

### ELISAs

All ELISA assays were carried out using commercially available affinity purified antibodies (Additional file 2). Indirect ELISAs were used to quantify protein expression, whereas sandwhich ELISAs were used to quantify protein-protein interactions. For indirect ELISAs, 40 μL of cleared cell lysate were absorbed onto a 96-well ELISA plate (NUNC) at room temperature for 1 hr. Following absorption, wells were washed 3X with 200 μL TBST at room temperature for 10 min. Wells were then blocked using 200 μL of StartingBlock™ Buffer (Thermo Scientific) according to the manufacturer’s directions. Following blocking, wells were washed 3X as described and 100 μL of TBST containing primary antibodies against one of twelve epitope tags (diluted 1:3000) were applied at room temperature for 1 hr. Wells were washed 3X and 100 μL of TBST containing HRP-conjugated secondary antibodies against the constant region of the detecting primary antibody (diluted 1:5000) were applied at room temperature for 1 hr. Wells were washed 3X and 100 μL of 1-Step Ultra TMB-ELISA reagent (Thermo Scientific) were applied, followed by 100 μL of 2 M sulfuric acid to stop the reaction. Absorbance was measured at 450 nm using a Tecan Safire 2 plate reader. For sandwich ELISAs, 100 μL of carbonate buffer containing antibodies against one of twelve epitope tags (diluted 1:500) were absorbed onto a 96-well ELISA plate at 4°C overnight. Following absorption, wells were washed 3X with 200 μL TBST at room temperature for 10 min and then blocked using 200 μL of StartingBlock™ Buffer. Following blocking, wells were washed 3X and 40 μL of cleared cell lysate applied at room temperature for 1 hr. Wells were washed 3X and 100 μL of TBST containing detecting primary antibodies against the second tag in the complex (diluted 1:3000) were applied at room temperature for 1 hr. The remaining washes, addition of the secondary HRP-conjugated antibodies (diluted 1:5000), addition of the TMB-ELISA reagent and of the sulfuric acid, as well as the absorbance measurements, were carried out as described in the indirect ELISA protocol. All ELISAs were performed in triplicate. Values were normalized for protein concentration and background. Control experiments included antibody drop-outs and the use of protein extracts lacking the epitope tags being assayed.

## Abbreviations

2ab: 2 antibiotic; A: Ampicillin; C: Chloramphenicol; K: Kanamycin; ORF: Open reading frame; RBS: Ribosome binding site; BBF: BioBricks™ Foundation; RFC: Request for comments; DIY: Do-it-yourself.

## Competing interests

The authors declare that they have no competing interests.

## Authors’ contributions

The experiments were designed by ML and JCA. Computational tools were developed by DD and JCA. DNA assemblies were performed by ML and JB. ELISAs were performed by ML and AA. The manuscript was drafted by ML and JCA. All authors read and approved the final manuscript.

## Supplementary Material

Additional file 1 Basic parts used for BglBricks-based 2ab assembly of bi-cistronic operons of Mediator complex. Click here for file

Additional file 2 Antibodies used in ELISA assays. Click here for file

Additional file 3 **Heat maps of protein concentration values obtained via indirect ELISAs. **Maps include all possible tags and tagging combinations in order to visualize expression patterns for each epitope-tagged ORF in all 528 bi-cistronic operons. The top map illustrates patterns of expression for Med7 located in the first ORF position and should be read from left to right. The bottom map illustrates patterns of expression for Med21 located in the second ORF position and should be read from top to bottom. Red color indicates high relative expression, whereas yellow color indicates low relative expression.The complete data set, including standard deviation values, are provided in Additional file 4. Click here for file

Additional file 4 Complete data set of protein concentration values obtained via indirect ELISAs from all 528 bi-cistronic operons. Click here for file
