# Supplementary material for: 2ab assembly: a methodology for automatable, high-throughput assembly of standard biological parts
Source: J Biol Eng. 2013 Jan 10;7:2. doi: 10.1186/1754-1611-7-2 (PMC3563576; doi:10.1186/1754-1611-7-2)
Supplement: Additional file 1 — Basic parts used for BglBricks-based 2ab assembly of bi-cistronic operons of Mediator complex. [file 1754-1611-7-2-S1.doc]

**Table S**1: Basic parts used for BglBricks-based 2ab assembly of bi-cistronic operons of Mediator complex

| **Basic part** | **Registry ID** | **Addgene ID** | **bp** | **Sequence** | **aa** | **Sequence** |
| --- | --- | --- | --- | --- | --- | --- |
| N-terminal tags |  |  |  |  |  |  |
| ntag-6XHis | J72016 | 26007 | 63 | GATCTCTACAACACCTAGCATCTAAGAAGTTACATATGGATTCTCACCATCATCACCATCACG | 9 | MDSHHHHHH |
| ntag-Avi | J72017 | 26008 | 83 | GATCTCACAAACTGATACAAACGGCGGGGAAATCATGGGCCTGAACGATATTTTTGAAGCGCAGAAAATTGAATGGCATGAAG | 16 | MGLNDIFEAQKIEWHE |
| ntag-E | J72018 | 26009 | 83 | GATCTCACATAGCGGCGCTAAATAATAGGGAGGCGATTCAATGGGTGCTCCGGTTCCATATCCAGATCCACTGGAACCACGTG | 14 | MGAPVPYPDPLEPR |
| ntag-FLAG | J72019 | 26010 | 72 | GATCTGGGACAATCCCGGTCTTTAAGAACAGGAATTACATGTCTGACGATTACAAGGATGACGACGACAAGG | 11 | MSDDYKDDDDK |
| ntag-HA | J72020 | 26011 | 75 | GATCTTAAGCACCCATAGAATACACACCTGGAGGAATAATGTCTTACCCATACGACGTCCCAGACTACGCTGGGG | 12 | MSYPYDVPDYAG |
| ntag-HSV | J72021 | 26012 | 80 | GATCTCGAACACCCATATCAATATTAAAGGGAAGGTAAGCATGCAGCCGGAGCTCGCACCAGAAGACCCGGAAGACTGCG | 13 | MQPELAPEDPEDC |
| ntag-Myc | J72022 | 26013 | 77 | GATCTACGCAATATCACACGGAACCGGAGGGCTAACTCGCATGTCTGAACAAAAACTCATCTCAGAAGAGGATCTGG | 12 | MSEQKLISEEDL |
| ntag-S | J72023 | 26014 | 87 | GATCTGAAAAGGAACCCACGCCAAGTCCAGGACACGCCATGAAAGAAACCGCTGCTGCTAAATTCGAACGTCAGCACATGGATTCCG | 16 | MKETAAAKFERQHMDS |
| ntag-Strep | J72024 | 26015 | 76 | GATCTTACAACTACACACTATCTCATACGGAGCCCTAACATGTCTGACACCTGGAGCCACCCGCAGTTCGAAAAAG | 12 | MSDTWSHPQFEK |
| ntag-T7 | J72025 | 26016 | 73 | GATCTGTACCAAATTAAAAAAATCAAAACGGGGTAAGATATGGCTTCTATGACTGGCGGTCAGCAAATGGGTG | 11 | MASMTGGQQMG |
| ntag-V5 | J72026 | 26017 | 84 | GATCTGAAGCAATATCTTCACGTATAAGGAGGTATTTCATGGGTAAGCCTATCCCTAACCCTCTGCTGGGTCTCGATTCTACGG | 15 | MGKPIPNPLLGLDST |
| ntag-VSV | J72027 | 26018 | 77 | GATCTCAATAGAACATTAGATCAGAACGCGAAAGGGAACAATGTACACCGATATAGAGATGAACAGGCTGGGAAAGG | 12 | MYTDIEMNRLGK |
| C-terminal tags |  |  |  |  |  |  |
| ctag-6XHis | J72028 | 26170 | 27 | GATCTCACCATCATCACCATCACTAAG | 6 | HHHHHH |
| ctag-Avi | J72029 | 26171 | 54 | GATCTGGCCTGAACGATATTTTTGAAGCGCAGAAAATTGAATGGCATGAATAAG | 15 | GLNDIFEAQKIEWHE |
| ctag-E | J72030 | 26172 | 48 | GATCTGGTGCTCCGGTTCCATATCCAGATCCACTGGAACCACGTTAAG | 13 | GAPVPYPDPLEPR |
| ctag-FLAG | K112603 | 26173 | 33 | GATCTGACTACAAGGATGACGACGACAAGTAAG | 8 | DYKDDDDK |
| ctag-HA | K112507 | 26174 | 39 | GATCTTACCCATACGACGTCCCAGACTACGCTGGGTAAG | 10 | YPYDVPDYAG |
| ctag-HSV | J72031 | 26175 | 45 | GATCTCAGCCGGAGCTCGCACCAGAAGACCCGGAAGACTGCTAAG | 12 | QPELAPEDPEDC |
| ctag-Myc | K112503 | 26176 | 39 | GATCTGAACAAAAACTCATCTCAGAAGAGGATCTGTAAG | 10 | EQKLISEEDL |
| ctag-S | J72032 | 26177 | 54 | GATCTAAAGAAACCGCTGCTGCTAAATTCGAACGTCAGCACATGGATTCCTAAG | 15 | KETAAAKFERQHMDS |
| ctag-Strep | J72033 | 26178 | 36 | GATCTACCTGGAGCCACCCGCAGTTCGAAAAATAAG | 9 | TWSHPQFEK |
| ctag-T7 | J72034 | 26179 | 42 | GATCTATGGCTTCTATGACTGGCGGTCAGCAAATGGGTTAAG | 11 | MASMTGGQQMG |
| ctag-V5 | J72035 | 26180 | 51 | GATCTGGTAAGCCTATCCCTAACCCTCTGCTGGGTCTCGATTCTACGTAAG | 14 | GKPIPNPLLGLDST |
| ctag-VSV | J72036 | 26181 | 42 | GATCTTACACCGATATAGAGATGAACAGGCTGGGAAAGTAAG | 11 | YTDIEMNRLGK |
| N-terminal ORFs |  |  |  |  |  |  |
| nMed7 | J72037 | 26182 | 324 | GATCTATGAAAAAATCTACCGAAAACGAATCTACCAACTACCAGTACAAAATCCAGGAACTGCGTAAACTGCTGAAATCTCTGCTGCTGAACTACCTGGAACTGATCGGTGTTCTGTCTATCAACCCGGACATGTACGAACGTAAAGTTGAAAACATCCGTACCATCCTGGTTAACATCCACCACCTGCTGAACGAATACCGTCCGCACCAGTCTCGTGAATCTCTGATCATGCTGCTGGAAGAACAGCTGGAATACAAACGTGGTGAAATCCGTGAAATCGAACAGGTTTGCAAACAGGTTCACGACAAACTGACCTCTTAAG | 105 | MKKSTENESTNYQYKIQELRKLLKSLLLNYLELIGVLSINPDMYERKVENIRTILVNIHHLLNEYRPHQSRESLIMLLEEQLEYKRGEIREIEQVCKQVHDKLTS |
| nMed21 | J72038 | 26183 | 405 | GATCTATGACCGACCGTCTGACCCAACTCCAAATTTGCCTGGACCAGATGACCGAACAGTTCTGCGCTACCCTGAACTACATCGACAAAAACCACGGTTTCGAACGTCTGACCGTTAACGAACCGCAGATGTCTGACAAACACGCTACCGTTGTTCCGCCGGAAGAGTTTTCTAACACTATTGACGAACTCTCCACCGACATCATCCTGAAAACCCGTCAGATCAACAAACTGATCGACTCTCTGCCAGGCGTTGACGTTTCCGCAGAGGAACAACTCCGTAAAATCGACATGCTCCAAAAAAAGCTCGTTGAAGTTGAAGACGAAAAAATCGAAGCTATCAAAAAAAAAGAAAAACTGCTGCGTCACGTTGACTCTCTGATCGAAGACTTCGTTGACGGTTAAG | 132 | MTDRLTQLQICLDQMTEQFCATLNYIDKNHGFERLTVNEPQMSDKHATVVPPEEFSNTIDELSTDIILKTRQINKLIDSLPGVDVSAEEQLRKIDMLQKKLVEVEDEKIEAIKKKEKLLRHVDSLIEDFVDG |
| C-terminal ORFs |  |  |  |  |  |  |
| cMed7 | J72039 | 26184 | 356 | GATCTCAGCTAAAATCAACGTATTAAGGCACTCCATAGCGATGAAAAAATCTACCGAAAACGAATCTACCAACTACCAGTACAAAATCCAGGAACTGCGTAAACTGCTGAAATCTCTGCTGCTGAACTACCTGGAACTGATCGGTGTTCTGTCTATCAACCCGGACATGTACGAACGTAAAGTTGAAAACATCCGTACCATCCTGGTTAACATCCACCACCTGCTGAACGAATACCGTCCGCACCAGTCTCGTGAATCTCTGATCATGCTGCTGGAAGAACAGCTGGAATACAAACGTGGTGAAATCCGTGAAATCGAACAGGTTTGCAAACAGGTTCACGACAAACTGACCTCTG | 105 | MKKSTENESTNYQYKIQELRKLLKSLLLNYLELIGVLSINPDMYERKVENIRTILVNIHHLLNEYRPHQSRESLIMLLEEQLEYKRGEIREIEQVCKQVHDKLTS |
| cMed21 | J72040 | 26185 | 432 | GATCTGAATACCCTACTAAGGACATTCGCCAGAAAATGACCGACCGTCTGACCCAACTCCAAATTTGCCTGGACCAGATGACCGAACAGTTCTGCGCTACCCTGAACTACATCGACAAAAACCACGGTTTCGAACGTCTGACCGTTAACGAACCGCAGATGTCTGACAAACACGCTACCGTTGTTCCGCCGGAAGAGTTTTCTAACACTATTGACGAACTCTCCACCGACATCATCCTGAAAACCCGTCAGATCAACAAACTGATCGACTCTCTGCCAGGCGTTGACGTTTCCGCAGAGGAACAACTCCGTAAAATCGACATGCTCCAAAAAAAGCTCGTTGAAGTTGAAGACGAAAAAATCGAAGCTATCAAAAAAAAAGAAAAACTGCTGCGTCACGTTGACTCTCTGATCGAAGACTTCGTTGACGGTG | 132 | MTDRLTQLQICLDQMTEQFCATLNYIDKNHGFERLTVNEPQMSDKHATVVPPEEFSNTIDELSTDIILKTRQINKLIDSLPGVDVSAEEQLRKIDMLQKKLVEVEDEKIEAIKKKEKLLRHVDSLIEDFVDG |
| Cleavage site |  |  |  |  |  |  |
| Tev | J72041 | 26186 | 27 | GATCTGAAAACCTCTATTTTCAAGGTG | 7 | ENLYFQG |
| Regulartory |  |  |  |  |  |  |
| pBad promoter | I0500 |  | 1244 | GATCTCTATGCTACTCCATCGAGCCGTCAATTGTCTGATTCGTTACCAATTATGACAACTTGACGGCTACATCATTCACTTTTTCTTCACAACCGGCACGGAACTCGCTCGGGCTGGCCCCGGTGCATTTTTTAAATACCCGCGAGAAATAGAGTTGATCGTCAAAACCAACATTGCGACCGACGGTGGCGATAGGCATCCGGGTGGTGCTCAAAAGCAGCTTCGCCTGGCTGATACGTTGGTCCTCGCGCCAGCTTAAGACGCTAATCCCTAACTGCTGGCGGAAAAGATGTGACAGACGCGACGGCGACAAGCAAACATGCTGTGCGACGCTGGCGATATCAAAATTGCTGTCTGCCAGGTGATCGCTGATGTACTGACAAGCCTCGCGTACCCGATTATCCATCGGTGGATGGAGCGACTCGTTAATCGCTTCCATGCGCCGCAGTAACAATTGCTCAAGCAGATTTATCGCCAGCAGCTCCGAATAGCGCCCTTCCCCTTGCCCGGCGTTAATGATTTGCCCAAACAGGTCGCTGAAATGCGGCTGGTGCGCTTCATCCGGGCGAAAGAACCCCGTATTGGCAAATATTGACGGCCAGTTAAGCCATTCATGCCAGTAGGCGCGCGGACGAAAGTAAACCCACTGGTGATACCATTCGCGAGCCTCCGGATGACGACCGTAGTGATGAATCTCTCCTGGCGGGAACAGCAAAATATCACCCGGTCGGCAAACAAATTCTCGTCCCTGATTTTTCACCACCCCCTGACCGCGAATGGTGAGATTGAGAATATAACCTTTCATTCCCAGCGGTCGGTCGATAAAAAAATCGAGATAACCGTTGGCCTCAATCGGCGTTAAACCCGCCACCAGATGGGCATTAAACGAGTATCCCGGCAGCAGGGGATCATTTTGCGCTTCAGCCATACTTTTCATACTCCCGCCATTCAGAGAAGAAACCAATTGTCCATATTGCATCAGACATTGCCGTCACTGCGTCTTTTACTGGCTCTTCTCGCTAACCAAACCGGTAACCCCGCTTATTAAAAGCATTCTGTAACAAAGCGGGACCAAAGCCATGACAAAAACGCGTAACAAAAGTGTCTATAATCACGGCAGAAAAGTCCACATTGATTATTTGCACGGCGTCACACTTTGCTATGCCATAGCATTTTTATCCATAAGATTAGCGGATCTTACCTGACGCTTTTTATCGCAACTCTCTACTGTTTCTCCATACCCG | n/a | n/a |
| Terminator | B0015 |  | 135 | GATCTCCAGGCATCAAATAAAACGAAAGGCTCAGTCGAAAGACTGGGCCTTTCGTTTTATCTGTTGTTTGTCGGTGAACGCTCTCTACTAGAGTCACACTGGCTCACCTTCGGGTGGGCCTTTCTGCGTTTATAG | n/a | n/a |
