# Supplementary material for: 2ab assembly: a methodology for automatable, high-throughput assembly of standard biological parts
Source: J Biol Eng. 2013 Jan 10;7:2. doi: 10.1186/1754-1611-7-2 (PMC3563576; doi:10.1186/1754-1611-7-2)
Supplement: Additional file 2 — Antibodies used in ELISA assays. [file 1754-1611-7-2-S2.doc]

**Table** S2: Antibodies used in ELISA assays

| **Antibody** | **Source** | **Catalog #** | **Immunogen** |
| --- | --- | --- | --- |
| goat anti-6X-His | Bethyl | A190-113A | HHHHHH-KLH |
| mouse anti-HIS-HRP | GenScript | A00612 | HHHHHH-KLH |
| rabbit anti-6X-His | GenScript | A00174 | HHHHHH-KLH |
| rabbit anti-Avi | GenScript | A00674 | GLNDIFEAQKIEWHE-KLH |
| goat anti-E | Bethyl | A190-132A | GAPVPYPDPLEPR-KLH |
| rabbit anti-E | GenScript | A00631 | GAPVPYPDPLEPR-KLH |
| mouse anti-FLAG | GenScript | A00013 | DYKDDDDK-KLH |
| goat anti-HA | GenScript | A00168 | YPYDVPDYA-KLH |
| rabbit anti-HA | Neomarkers | rb-1438-PO | CYPYDVPDYASL |
| goat anti-HSV | Bethyl | A190-136A | SQPELAPEDPED-KLH |
| rabbit anti-HSV | GenScript | A00624 | QPELAPEDPED-KLH |
| mouse anti-Myc | GenScript | A00704 | EQKLISEEDL-KLH |
| rabbit anti-Myc | GenScript | A00172 | EQKLISEEDL-KLH |
| goat anti-S | Bethyl | A190-134A | KETAAAKFERQHMDS-KLH |
| rabbit anti-S | GenScript | A00625 | KETAAAKFERQHMDS-KLH |
| rabbit anti-Strep | GenScript | A00626 | NWSHPQFEK-KLH |
| rabbit anti-Strep-HRP | GenScript | A00875 | NWSHPQFEK-KLH |
| goat anti-T7 | Abcam | ab1271 | MASMTGGQQMG-KLH |
| rabbit anti-T7 | GenScript | A00622 | MASMTGGQQMG-KLH |
| goat anti-V5 | GenScript | A00099 | GKPIPNPLLGLDST-KLH |
| rabbit anti-V5 | GenScript | A00623 | GKPIPNPLLGLDST-KLH |
| goat anti-VSV | Bethyl | A190-130A | YTDIEMNRLGK-KLH |
| rabbit anti-VSV | GenScript | A00199 | YTDIEMNRLGK-KLH |
| rabbit anti-Goat-HRP | Abcam | ab6741 | goat IgG whole molecule |
| rabbit anti-Mouse-HRP | Abcam | ab6728 | mouse IgG whole molecule |
| goat anti-Rabbit-HRP | Abcam | ab6721 | rabbit IgG whole molecule |
