# Supplementary figures and images for: 2ab assembly: a methodology for automatable, high-throughput assembly of standard biological parts
Source: J Biol Eng. 2013 Jan 10;7:2. doi: 10.1186/1754-1611-7-2 (PMC3563576; doi:10.1186/1754-1611-7-2)

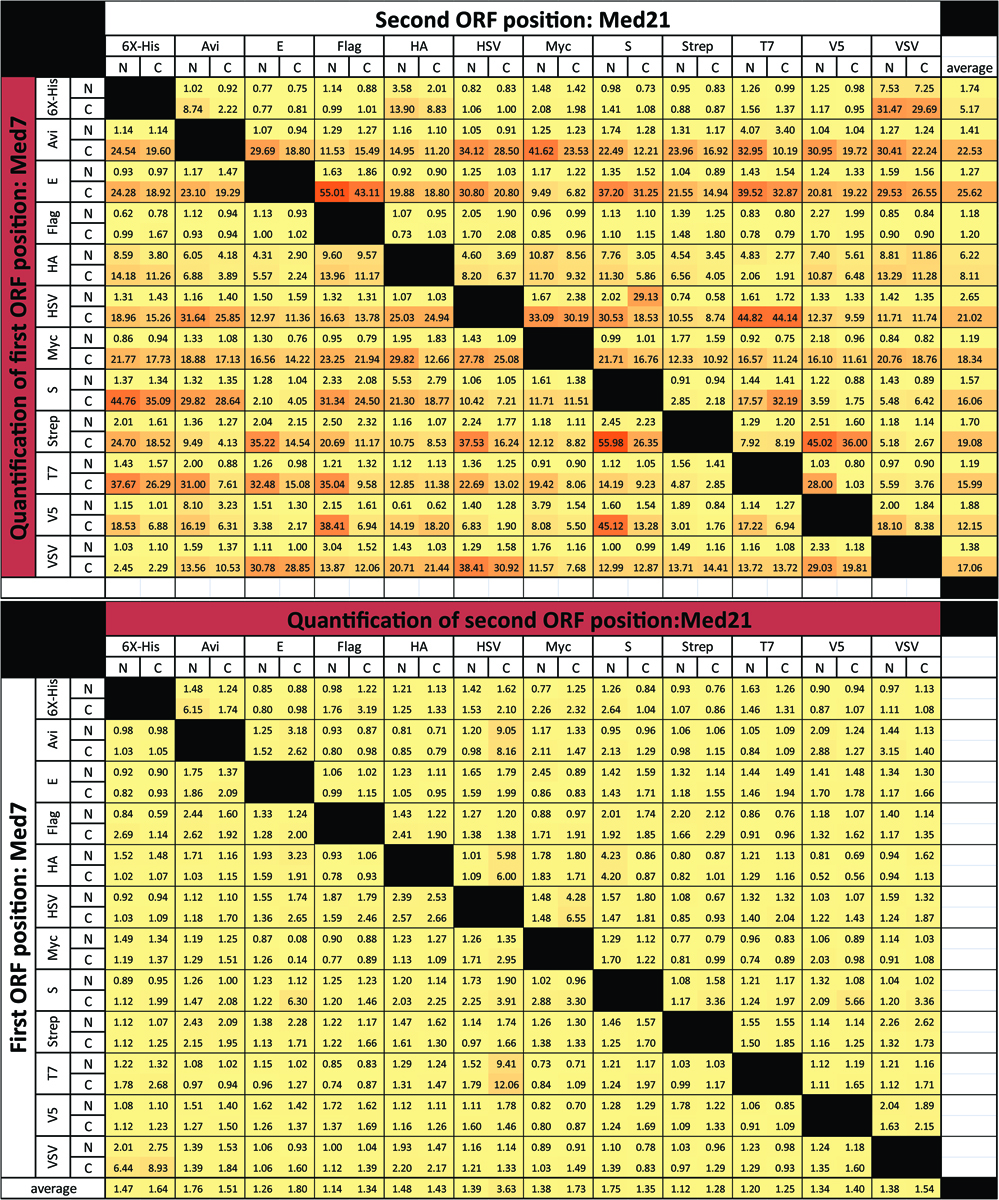

Supplement: Additional file 3 — Heat maps of protein concentration values obtained via indirect ELISAs. Maps include all possible tags and tagging combinations in order to visualize expression patterns for each epitope-tagged ORF in all 528 bi-cistronic operons. The top map illustrates patterns of expression for Med7 located in the first ORF position and should be read from left to right. The bottom map illustrates patterns of expression for Med21 located in the second ORF position and should be read from top to bottom. Red color indicates high relative expression, whereas yellow color indicates low relative expression.The complete data set, including standard deviation values, are provided in Additional file 4. [file 1754-1611-7-2-S3.jpeg]
